# The Pension Minister at Leicester

**Published:** 1918-08-10

**Authors:** 


### THE PENSIONS MINISTER AT LEICESTER.

### An Interesting Day's Work.

The visit of Mr. John Hodge, M.P., was looked forward to with great interest. Ostensibly his visit was to open the Leicester Frith Home for Neurasthenic and Shell-shocked Soldiers, established by the Mayor of Leicester (Alderman Jonathan North, J.P.) from his Wounded Heroes Fund. Opportunity was, however, found to hold a useful conference with those responsible for the administration of the Leicester and Leicestershire War Pensions Committee, to pay visits of inspection to the local hospitals undertaking the treatment of wounded soldiers, and for Mr. Hodge to address a mass meeting in the De Montfort Hall.

The Opening of the Home: Generous Gifts.

The Mayor presided at the formal opening of the Home, which, he announced, was, with the surrounding grounds, secured at an annual rental from the Corporation, and was equipped, at the entreaty of the Ministry of Pensions, to provide a Home of Recovery for wounded and shell-shocked soldiers. £8,000 had been spent in building improvements and additions, and in overhauling the Home and equipping and furnishing it. When completed, the accommodation would be for 100 patients. Preference would be given when possible to Leicester and Leicestershire sailors and soldiers, but the committee were prepared to receive applications from men in any part of the kingdom while there was available accommodation. In the equipment of the Home material help had been received from teachers and scholars of the local elementary schools, who had collected £1,500; from the West-End Association for the Entertainment of Wounded Soldiers, who had borne the cost of fitting up the gymnasium and had provided a most useful library; from the Leicester Musical Society, who had contributed £100 from their annual concert; and from Mr. T. G. Hunt and friends, who had provided a handsome piano and many pictures. He announced with pleasure an offer by the Leicester Association of Engineers to equip the Home with such machinery and appliances as the medical officer might deem necessary, to the cost of £1,000.

With regard to the provision of an Orthopædic Centre, he regretted the setback to the scheme, which would have been completed by this time, if the necessary sanction could have been obtained from the central authorities. With the assistance of the right honourable gentleman who had favoured them with his presence and with his powerful help, they hoped to be able to remove this embargo.

Orthopædics.

The Duke of Rutland expressed the opinion that there was no work which the Mayor of Leicester had done in his four years of office which would be held in more kindly and affectionate remembrance by his fellow-townsmen and by the county than the great work he had inaugurated and carried out at the Home. It would be held for all time in grateful memory by a vast number of suffering men, and he thought no greater recognition of anyone's work at this time could be made than that he should be remembered for having done something to help those gallant fellows who had been fighting abroad.

Lieutenant-General Maxwell (G.O.C. Northern Command) gave expression to the opinion that one of the brightest features of the war was the ready and continued help which all communities were giving to aid sailors and soldiers suffering from wounds and sickness. Another bright feature was the help of nurses and the medical profession, who had rendered magnificent service at home and abroad.

A handsome souvenir emblematical of Victory and Peace having been presented by Mr. S. Perkins Pick, the architect, Mr. John Hodge declared the building open. He voiced the opinion that the payment of pensions was a small matter compared with the restoration to health and the reinstatement of men in civil life. The Home he now opened was not a charitable institution. Its upkeep was paid for out of State funds, although the Home and its equipment had been provided by the generosity of friends in Leicester and the county. He was prepared to accept similar gifts at any time and anywhere. If they could get premises like those he now opened they could get them equipped and in use while the Treasury was thinking whether it could allow new premises to be built. Never in the history of the country had medical work and science stood so high in estimation. He hoped institutions similar to the present one would soon be scattered all over the country, so that wounded and injured men could have treatment near to their homes. He hoped also that the heart of the Mayor would be gladdened by the opening of an Orthopædic Centre in Leicester.

Thanks having been accorded to the Pensions Minister on the motion of the High Sheriff, Mr. T. Fielding Johnson, jun., J.P., opportunity was afforded to the visitors of looking over the premises and of inspecting the handiwork of the patients in residence.

Friday was spent by the Pensions Minister in visiting the 5th Northern General Hospital, the Royal Infirmary, and the Gilroes Hospital (Corporation).

At the Royal Infirmary he was met by representative governors, and Mr. Fielding Johnson, jun., the chairman of the board, renewed the offer of the governors and of the Mayor of Leicester to build and equip an Orthopædic Centre at the institution. The board had already secured adjoining sites at a cost of several thousand pounds for the proposed structure, but they did not feel justified in expending the money of the institution on temporary buildings as suggested by the Ministry of Munitions. Figures were presented to Mr. John Hodge showing that since the month of March nearly 1,200 attendances had been made by discharged soldiers in the massage and electrical treatment department, which was at present dealing with approximately 350 cases per week, and was totally inadequate for the treatment which they were called upon to give. About 120 patients had also received in-patients' treatment in the civil wards, and it would be much more satisfactory from all points of view if these could be treated in the proposed in-patients' department. Mr. John Hodge expressed his gratitude for the renewed offer, which he promised to bring to the notice of the Chancellor of the Exchequer to see if something could not be done to give effect to the generous wishes of the governors. A visit of inspection was paid to the existing department, where Mr. Hodge saw the treatment which was being carried out and inspected the equipment. He was subsequently shown the site for the intended structure, which he described as eminently satisfactory.

It is now hoped that the proposed department will receive reconsideration from the Government, and steps be taken to remove the embargo and so remedy a state of affairs which is causing much dissatisfaction to the Local Pensions Committees and is detrimental to the interests of wounded sailors and soldiers.

